# Supplementary material for: Distinct p21 dynamics drive alternative routes to whole-genome duplication through a common CDK4/6-dependent polyploid G0 state
Source: bioRxiv. 2026 Jan 15:2026.01.15.699545. Preprint. [Version 1] doi: 10.64898/2026.01.15.699545 (PMC12871375; doi:10.64898/2026.01.15.699545)
Supplement: 1 [file NIHPP2026.01.15.699545V1-supplement-1.pdf]

bioRxiv preprint doi: https://doi.org/10.64898/2026.01.15.699545; this version posted January 15, 2026. The copyright holder for this preprint (which was not certified by peer review) is the author/funder, who has granted bioRxiv a license to display the preprint in perpetuity. It is made available under aCC-BY 4.0 International license.

Cell Medium Composition				
cell_line	Cellosaurus Accession	Base medium	Supplements	Antibiotics
MDA-MB-231	CVCL_0062	DMEM (Gibco, cat. 1965)	10% fetal bovine serum (FBS; Gibco, cat. 26140)	1% penicillin–streptomycin (P/S; Gibco, cat. 15070-063)
MDA-MB-453	CVCL_0418	DMEM (Gibco, cat. 1965)	10% fetal bovine serum (FBS; Gibco, cat. 26140)	
MCF7	CVCL_0031	DMEM (Gibco, cat. 1965)	10% fetal bovine serum (FBS; Gibco, cat. 26140)	
T47D	CVCL_0553	RPMI-1640 medium (Gibco, cat. 11875)	10% fetal bovine serum (FBS; Gibco, cat. 26140)	
MDA-MB-134IV	CVCL_0617	1:1 mixture of high-glucose DMEM (Gibco, cat. 1965) and Leibovitz's L-15 medium (Sigma, cat. L5520)	10% fetal bovine serum (FBS; Gibco, cat. 26140)	
SUM-44PE	CVCL_3424	DMEM/F-12 medium (Gibco, cat. 11330)	2% charcoal-stripped serum (CSS; Gibco, cat. A33821-01), 5 μg/mL insulin (Gibco, cat. 12585-014), 1 μg/mL hydrocortisone (Sigma, cat. H6909), 5 mM ethanolamine (Sigma, cat. E0135), 5 μg/mL transferrin (Sigma, cat. T2252), 10 nM triiodothyronine (T3; Sigma, cat. T5516), 8.7 ng/mL sodium selenite (Sigma, cat. S9133)	
BCK4	CVCL_A9A5	MEM (Gibco, cat. 11095)	1% MEM non-essential amino acids (NEAA; Gibco, cat. 11140), 1 nM insulin (Gibco, cat. 12585-014), 5% FBS (Gibco, cat. 26140 or Gemini, cat. 100-119)	
MCF10A	CVCL_0598	DMEM/F-12 medium (Gibco, cat. 11330)	10 μg/mL insulin (Gibco, cat. 12585-014), 200 ng/mL epidermal growth factor (EGF; Sigma, cat. E9644-0.2MG), 20 ng/mL cholera toxin (Sigma, cat. C8052-5MG), 0.5 mg/mL hydrocortisone (Sigma, cat. H6909), and 5% horse serum (Gibco, cat. 16060-112)	

Table S1

bioRxiv preprint doi: https://doi.org/10.64898/2026.01.15.699545; this version posted January 15, 2026. The copyright holder for this preprint (which was not certified by peer review) is the author/funder, who has granted bioRxiv a license to display the preprint in perpetuity. It is made available under aCC-BY 4.0 International license.

Reagent or Resource	Source	Identifier	Fluorescent Dye	Dilution
Anti-53BP1 antibody	Abcam	Cat# ab222232	AF750	1:50
Alexa Fluor® 647 Anti-Aurora B antibody	Abcam	Cat# ab197614	AF647	1:100
Anti-β-Catenin−Cy3 antibody	Sigma-Aldrich	Cat# C7738	AF555	1:100
Alexa Fluor® 488 Anti-Cdc25C antibody	Abcam	Cat# ab205425	AF488	1:100
Human/Mouse/Rat CDK2 Antibody	R&D Systems	Cat# AF4654	AF750	1:100
Anti-Cdk4 antibody	Abcam	Cat# ab213216	AF750	1:100
Alexa Fluor® 647 Anti-Cdk6 antibody	Abcam	Cat# ab198946	AF647	1:200
Alexa Fluor® 647 Anti-CDT1/DUP antibody	Abcam	Cat# ab211857	AF647	1:100
Alexa Fluor® 647 Anti-c-Myc antibody	Abcam	Cat# ab190560	AF647	1:100
Alexa Fluor® 555 Anti-Cyclin A2 antibody	Abcam	Cat# ab217731	AF555	1:300
Alexa Fluor® 555 Anti-Cyclin B1 antibody	Abcam	Cat# ab214381	AF555	1:200
Anti-Cyclin B2/CCNB2 antibody	Abcam	Cat# ab250841	AF750	1:50
Alexa Fluor® 555 Anti-Cyclin D1 antibody	Abcam	Cat# ab203448	AF555	1:100
Anti-Cyclin D3/CCND3 antibody	Abcam	Cat# ab245734	AF750	1:100
Alexa Fluor® 647 Anti-Cyclin E1 antibody	Abcam	Cat# ab194069	AF647	1:100
Alexa Fluor® 488 Anti-Cyclin E2 antibody	Abcam	Cat# ab207336	AF488	1:100
Purified anti-E2F1 Antibody	Biolegend	Cat# 606051	AF750	1:100
Human Cyclin A1 Antibody	R&D Systems	Cat# MAB7046	AF555	1:100
E-Cadherin (24E10) Rabbit mAb	Cell Signaling Technology	Cat# 96743	AF555	1:100
fzr Antibody	Santa Cruz	Cat# sc-56312	AF647	1:100
Alexa Fluor® 555 Anti-Estrogen Receptor alpha antibody	Abcam	Cat# ab279333	AF555	1:100
Alexa Fluor® 488 Anti-ErbB2 / HER2 antibody	Abcam	Cat# ab225509	AF488	1:100
Alexa Fluor® 488 Anti-Hes1 antibody	Abcam	Cat# ab196328	AF488	1:100
Na,K-ATPase α1 (D4Y7E) Rabbit mAb	Cell Signaling Technology	Cat# 99935	AF750	1:100
Anti-p130 antibody	Abcam	Cat# ab247453	AF750	1:100
Alexa Fluor® 647 Anti-CDKN2A/p16INK4a antibody	Abcam	Cat# ab192054	AF647	1:100
p21 Waf1/Cip1 (12D1) Rabbit mAb	Cell Signaling Technology	Cat# 8587	AF647	1:100
Anti-p27 KIP 1 antibody	Abcam	Cat# ab206927	AF750	1:50
p38 MAPK (D13E1) XP® Rabbit mAb	Cell Signaling Technology	Cat# 54470	AF555	1:100
Alexa Fluor® 647 Anti-p53 antibody	Abcam	Cat# ab224942	AF647	1:100
NF-κB p65 (D14E12) XP® Rabbit mAb	Cell Signaling Technology	Cat# 49445	AF488	1:100
Phospho-Akt (Ser473) (D9E) XP® Rabbit mAb	Cell Signaling Technology	Cat# 4075	AF647	1:100
Anti-ATR (phospho T1989) antibody	Abcam	Cat# ab289363	AF750	1:200
Anti-Cdc6 (phospho S54) antibody	Abcam	Cat# ab247402	AF750	1:100
PCNA (D3H8P) XP® Rabbit mAb	Cell Signaling Technology	Cat# 82968	AF647	1:100
Ki-67 (8D5) Mouse mAb	Cell Signaling Technology	Cat# 81240	AF647	1:500
Phospho-p44/42 MAPK (Erk1/2) (Thr202/Tyr204) (D13.14.4	Cell Signaling Technology	Cat# 45899	AF647	1:100
Alexa Fluor® 555 Anti-gamma H2A.X (phospho S139) antib	Abcam	Cat# ab206900	AF555	1:100
Phospho-Histone H3 (Ser10) (D7N8E) XP® Rabbit mAb	Cell Signaling Technology	Cat# 47398	AF488	1:100
Alexa Fluor® 488 Anti-PLK1 antibody	Abcam	Cat# ab223901	AF488	1:100
Anti-p130 (phospho S672) antibody	Abcam	Cat# ab284755	AF750	1:100
Progesterone Receptor A/B (D8Q2J) XP® Rabbit mAb	Cell Signaling Technology	Cat# 18444	AF750	1:100
Phospho-Rb (Ser807/811) (D20B12) XP® Rabbit mAb	Cell Signaling Technology	Cat# 8974	AF647	1:100
Phospho-S6 Ribosomal Protein (Ser240/244) (D68F8) XP® 1	Cell Signaling Technology	Cat# 5018	AF488	1:100
Rb (4H1) Mouse mAb	Cell Signaling Technology	Cat# 61121	AF750	1:100
Skp2 (D3G5) XP® Rabbit mAb	Cell Signaling Technology	Cat# 2652	AF647	1:100
Wee1 (D10D2) Rabbit mAb	Cell Signaling Technology	Cat# 13084	AF555	1:200
YAP (D8H1X) XP® Rabbit mAb	Cell Signaling Technology	Cat# 53921	AF555	1:100

Table S2
